# Contributions to the systematics of New World macro-moths VI

**DOI:** 10.3897/zookeys.527.6803

**Published:** 2015-10-15

**Authors:** B. Christian Schmidt, J. Donald Lafontaine

**Affiliations:** 1 Canadian National Collection of Insects, Arachnids, and Nematodes, Biodiversity Program, Agriculture and Agri-Food Canada, KW Neatby Bldg., 960 Carling Ave., Ottawa, Ontario, Canada K1A 0C6

This special issue of ZooKeys is the sixth volume in the “*Contributions*” series, dedicated to disseminating systematics research on the Noctuoidea, Geometroidea, and other macro-moth groups. Previous volumes were published in May 2009 (volume I, ZooKeys 9), March 2010 (volume II, ZooKeys 39), November 2011 (volume III, ZooKeys 149), February 2013 (volume IV, ZooKeys 264) and June 2014 (volume V, ZooKeys 421) (see Schmidt and Lafontaine 2009, 2010, 2011, 2013, 2014). Authors interested in contributing to future “*Contributions*” volumes are encouraged to contact us.

In the current volume, eight authors contributed six manuscripts on North American taxa in the Erebidae and Noctuidae. In addition to 55 taxonomic changes, ten new taxa are described from North America, including six species of *Doryodes* from the eastern United States (*Doryodes desoto* Lafontaine & Sullivan, **sp. n.**; *Doryodes okaloosa* Sullivan & Lafontaine, **sp. n.**; *Doryodes fusselli* Sullivan & Lafontaine, **sp. n.**; *Doryodes reineckei* Sullivan & Lafontaine, **sp. n.**; *Doryodes broui* Lafontaine & Sullivan, **sp. n.**; and *Doryodes latistriga* Sullivan & Lafontaine, **sp. n.**), two species from the western United States (*Ogdoconta margareta* Crabo, **sp. n.** and *Lacinipolia dimocki* Schmidt, **sp. n.**), and two new genera (*Paraseptis* Mustelin & Crabo, **gen. n.** and *Viridiseptis* Mustelin & Crabo, **gen. n.**). All updates and corrections to the Check List of North American Noctuoidea (Lafontaine and Schmidt 2010) since the last update in 2013 are summarized.
